# 3D Biomimetic Magnetic Structures for Static Magnetic Field Stimulation of Osteogenesis

**DOI:** 10.3390/ijms19020495

**Published:** 2018-02-07

**Authors:** Irina Alexandra Paun, Roxana Cristina Popescu, Bogdan Stefanita Calin, Cosmin Catalin Mustaciosu, Maria Dinescu, Catalin Romeo Luculescu

**Affiliations:** 1 Center for Advanced Laser Technologies (CETAL), National Institute for Laser, Plasma and Radiation Physics, Magurele, RO-077125 Bucharest, Romania; irina.paun@inflpr.ro (I.A.P.); bogdan.calin@inflpr.ro (B.S.C.); 2 Faculty of Applied Sciences, University Politehnica of Bucharest, RO-060042 Bucharest, Romania; 3 Horia Hulubei National Institute for Physics and Nuclear Engineering IFIN-HH, Magurele, RO-077125 Bucharest, Romania; roxpopescu@yahoo.co.uk (R.C.P.); cosmin@nipne.ro (C.C.M.); 4 Faculty of Applied Chemistry and Materials Science, University Politehnica of Bucharest, RO-011061 Bucharest, Romania; 5 National Institute for Laser, Plasma and Radiation Physics, Magurele, RO-077125 Bucharest, Romania; dinescum@nipne.ro

**Keywords:** static magnetic field stimulation, 3D biomimetic structures, bone cell growth and differentiation

## Abstract

We designed, fabricated and optimized 3D biomimetic magnetic structures that stimulate the osteogenesis in static magnetic fields. The structures were fabricated by direct laser writing via two-photon polymerization of IP-L780 photopolymer and were based on ellipsoidal, hexagonal units organized in a multilayered architecture. The magnetic activity of the structures was assured by coating with a thin layer of collagen-chitosan-hydroxyapatite-magnetic nanoparticles composite. In vitro experiments using MG-63 osteoblast-like cells for 3D structures with gradients of pore size helped us to find an optimum pore size between 20–40 µm. Starting from optimized 3D structures, we evaluated both qualitatively and quantitatively the effects of static magnetic fields of up to 250 mT on cell proliferation and differentiation, by ALP (alkaline phosphatase) production, Alizarin Red and osteocalcin secretion measurements. We demonstrated that the synergic effect of 3D structure optimization and static magnetic stimulation enhances the bone regeneration by a factor greater than 2 as compared with the same structure in the absence of a magnetic field.

## 1. Introduction

Nowadays, the term tissue engineering is frequently used in order to address the solutions for tissue replacement following an accident, surgical excision or organ loss of function. The concept refers to the “development of biological substitutes that restore, maintain or improve the tissue function” [1]. Starting from this concept, the strategy of developing 3D biomimetic structures that simulate the architecture of the natural tissue has evolved into implementing specific properties to the structures for stimulating the cells growth, development and differentiation into functional tissue.

Bone tissue engineering has been one of the hot topics in biomaterials science, due to the increased need of tissue replacement in traumas, tumor excision, skeletal abnormalities or resection. A successful regeneration of this tissue depends on the interface interactions that take place between the osteoblasts and the 3D structure, on the ability of cells penetration, growth and development inside the construct, as well as on their access to growth factors and nutrients.

Engineered materials for bone implants make use of different physical external stimuli, such as magnetic, electric or mechanic, in order to accelerate the repair and regeneration in the affected tissue [2]. In particular, magnetic field stimulation has been proved to promote the integration of the implant, to determine an increased bone density of the newly developed tissue, by increasing the calcium content, thus promoting a more rapid and better healing of the affected bone [2,3]. Static magnetic fields were found to accelerate the proliferation, migration, orientation or differentiation of osteoblast-like cells [4,5,6,7,8,9], and to induce the osteogenic differentiation in bone marrow-derived mesenchymal stem cells [10,11,12]. These effects can be correlated to the fact that the cell membrane has diamagnetic properties and the membrane flux can be modified by exposure to static magnetic field [13,14,15]. In addition, the extracellular matrix proteins have diamagnetic properties, their structure and orientation being also affected by static magnetic fields [16]. Weak static magnetic or pulsed electromagnetic fields are also effective stimuli for bone fracture healing, spinal fusion, bone ingrowths into ceramics in animal models. Strong static magnetic fields of 5–10 T were also found to regulate the orientation of matrix proteins and cells in vitro and in vivo [12,13,16].

Researchers have previously studied the effects of static magnetic fields on cells cultured on different non-magnetic substrates [5], but implantable structures incorporating superparamagnetic nanoparticles have gained significantly more interest [2,17,18,19]. Owing to their intrinsic magnetic properties, they have the ability to improve the adhesion and growth of cells, even in the absence of an external magnetic field [20,21,22,23,24]. In particular, composites containing magnetic nanoparticles (MNPs) integrated in various matrices showed significant potentials as bone substitutes. Ceramic composites containing super-paramagnetic nanoparticles, hydroxyapatite and tricalcium phosphate had good biocompatibility with the bone cells, and the presence of the nanoparticles did not affect the function of the bone morphological protein binding to the composites [25]. Magnetic, biodegradable Fe_3_O_4_/chitosan/poly (vinyl alcohol) nanofibrous films fabricated by electrospinning, with average fiber diameters ranging from 230 to 380 nm and porosity of 83.9–85.1%, facilitated the osteogenesis in MG-63 human osteoblast-like cells [26]. Magnetic hydroxyapatite coatings with oriented nanorod arrays using magnetic bioglass coatings as sacrificial templates were also fabricated and used as substrates for bone growth. To date, the magnetic implantable structures have been fabricated either by dip-coating conventional structures in aqueous ferrofluids containing iron oxide nanoparticles coated with various biopolymers or by direct nucleation of biomimetic phase and super-paramagnetic nanoparticles on self-assembling collagen fibers [27].

The major goal of this work is to accelerate the osteogenesis via the synergic effect of magnetic 3D structures in response to weak static magnetic fields. For this, we designed and fabricated novel complex 3D structures with unitary elements that mimic the shape of native osteoblast-like cells, using laser direct writing via two photons polymerization (LDW via TPP) method. For large-scale use of these structures, as, for example, future in vivo studies with envisaged outcome to be translated into clinics, an important point is that the material designs need to be reproduced with exactly the same dimensions for every application. LDW via TPP is a method with great advantages in manufacturing arbitrary three dimensional (3D) micro/nanostructures of polymers, hybrid materials, organically modified ceramics (Ormocer), and metals with high reproducibility and sub diffraction-limit resolution down to 100 nm [28]. The high reproducibility of the 3D structures is mostly provided by the fact that LDW via TPP uses three-dimensional computer aided design. LDW via TPP is a layer-by-layer method where every layer is stacked up by voxels. The two-photon solidified small volume elements called voxels are quite reproducible, their size fluctuating within less than 8% (from the actual sizes down to 1 µm depending on the multicomponent optical objective used for laser focusing). The micro/nanostructures are formed by the stack of the voxels, so the resolution and spatial arrangement of the voxels play an important role in fabricating high precise structures. The high accuracy and reproducibility of the 3D structures implies the use of small voxels and tight arrangement. Owing to these characteristics, LDW via TPP is generally recognized as having high reproducibility and fidelity in obtaining 3D structures with complex architectures [28]. After fabrication, we identified the 3D structures that provided the best micro-environmental conditions for cell attachment and growth and favored the cells interconnections in complex 3D architectures, similar with those encountered in vivo. Then, we provided the structures with the function of responding to applied static magnetic fields, by coating them with collagen (Col)-chitosan (Chi)-hydroxyapatite (HA)-magnetic nanoparticles (MNPs) composite. Thus, we obtained new biomimetic magnetically responsive structures aiming to accelerate the osteogenic response in vitro in osteoblast-like cells, when exposed to static magnetic fields. We stimulated the cell-seeded structures using a range of weak magnetic fields intensities (between 100–250 mT) that was not investigated in previous studies and we provide evidence of their osteogenic effect.

## 2. Results and Discussion

### 2.1. Structures Optimization

**Figure 1:**
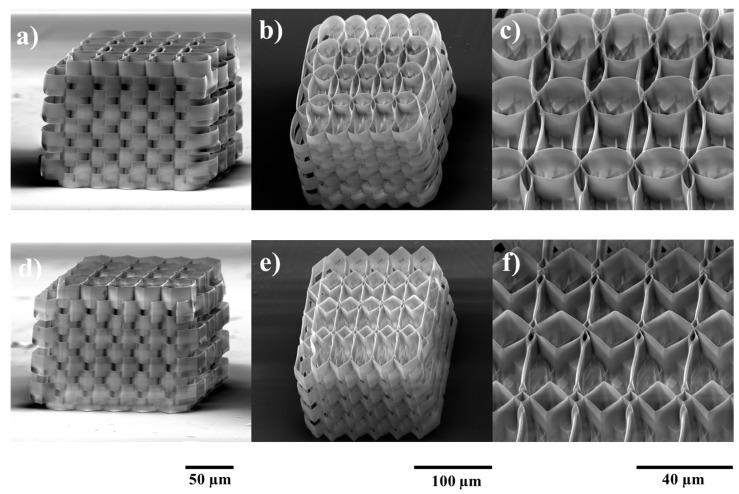
SEM micrographs of ellipsoidal (upper panel) and hexagonal (lower panel) multilayered 3D structures produced by LDW (laser direct writing) via TPP (two photon polymerization) of IP-L780 photopolymer. (**a**,**d**) Side overviews; (**b**,**e**) Tiled overviews; (**c**,**f**) Closer, tilted views of the structures.

The fabricated structures closely followed the design, yet not perfectly, as there are some geometry variations determined by intrinsic material properties and development methodology (Figure 1). Voxel height accounts for stronger overlap on the *Z* axis. This results in better structural integrity, albeit along with lowering porosity and potentially hindering cell migration due to smaller transfer windows throughout the structure.

Variations at the edge of the structure were determined by both material properties and development methodology. During irradiation, a series of chemical reactions result in the formation of polymeric chains. The density of the resulting polymer is slightly higher compared to non-irradiated material. As such, there is mechanical tension of various strengths throughout the irradiated volume. Moreover, until the sample is developed and dried, the polymer possesses higher flexibility, adherence and surface charges. This results in the welding of neighboring structures which, in combination with other effects of the irradiation (mechanical tension and surface charges), induces small variations of geometry at every contact point. After development, during the drying phase of the sample, surface tension of the evaporating developer can also induce deformation of the still-flexible polymer. This can be observed in Figure 1a. Apart from edge effects, the structure presents high stability and integrity due to the high number of contact points. Negligible differences from the design can still be observed at contact points, yet these are not considered variations as they are well reproduced throughout the whole structure.

The exponential overlap is designed for the *Y*-axis. The structure is designed to have 4 rows with respect to this axis. As such, on the *Y*-axis, there are 3 contact points with different overlaps. This results in rows with different spatial parameters (such as porosity) in the same structure. Its purpose is to determine the optimal overlap for cell growth throughout the entire volume of the structure.

**Figure 2:**
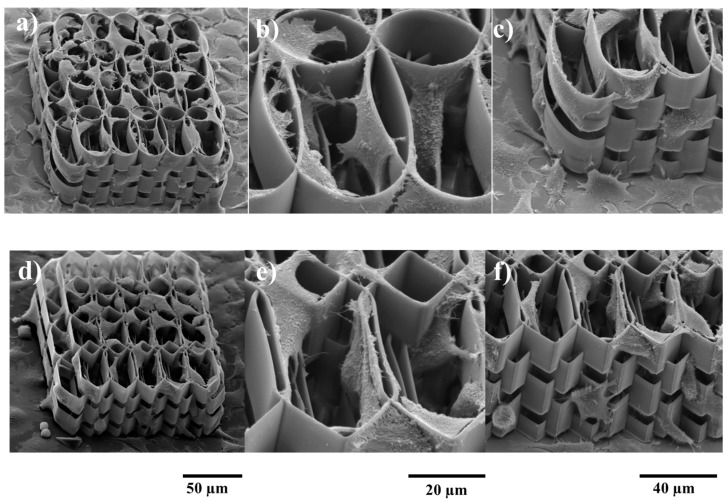
SEM (scanning electron microscopy) micrographs of MG-63 osteoblast like cells seeded for two days on ellipsoidal (upper panel) and hexagonal (lower panel) multilayered 3D structures. (**a**,**d**) Overviews of cells attachment on the structures; (**b**,**e**) Cells penetrating inside the structures; (**c**,**f**) Cells growing on the lateral walls of the structures.

Figure 2 shows the morphology of the cells during the first days of culture on the ellipsoidal and hexagonal multilayered 3D structures. The aspect is heterogeneous, depending on the movement of the cells and their affinity for the area in which they have settled (morphology is given by the number and position of the attachment points on the structure). In the detailed pictures (Figure 2b,c) it is evidenced the tendency of the cells to migrate into the interior of the structure, to climb onto the lateral walls and to travel through the inner part of the structure, where the specific surface is higher and thus the higher the number of attachment points. We can also observe a tendency of the osteoblast-like cells surface density to increase with higher overlap. For medium and low overlaps of the ellipsoidal and hexagonal elements, the cells seem to have better migrated through whole volume of the scaffold. While this distribution is found for both the elliptic and the hexagonal structures, we observe a higher density for the elliptic version. This is determined by the shape of the edges, as the cells seem to attach better on rounded edges rather than straight ones.

**Figure 3:**
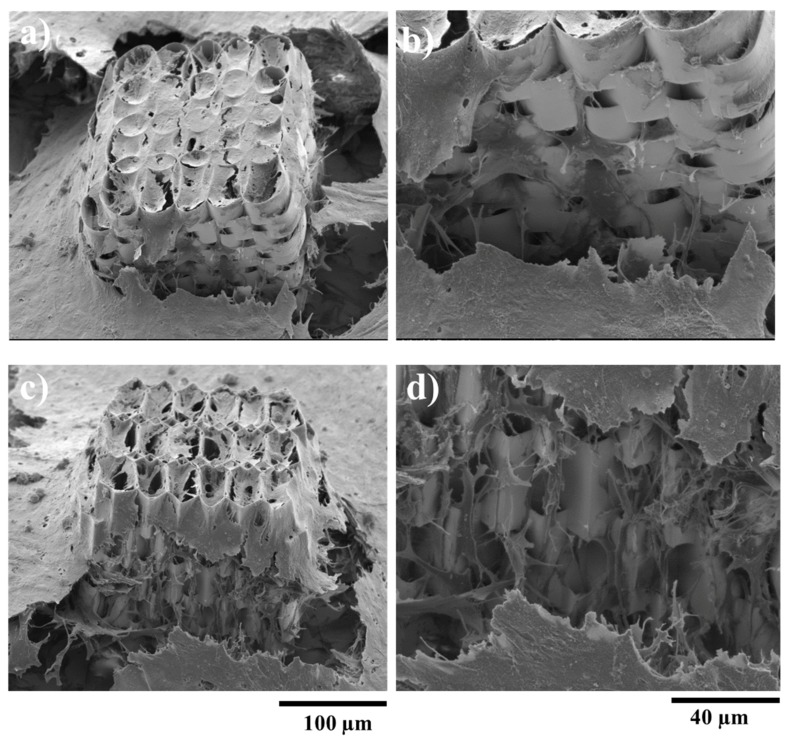
SEM (scanning electron microscopy) micrographs of MG-63 osteoblast like cells growing on ellipsoidal (upper panel) and hexagonal (lower panel) multilayered 3D structures with optimum horizontal arrangement, after 7 days in culture. (**a**,**c**) Overviews; (**b**,**d**) Cells growing on the lateral walls of the structures.

SEM (scanning electron microscopy) images for MG-63 cells cultured during 7 days showed the high potential of the structures to support the cells growth. It can be clearly seen that the cells invaded both the ellipsoidal and hexagonal structures, on the outside as well as on the lateral walls (Figure 3b). In case of the ellipsoidal structure, the cells have a circular shape, given by the growth support, forming a continuous layer on the top surface of the scaffold (Figure 3a). The lateral walls show few cells attached to the exterior of the ellipsoidal elements, the attachment points for the osteoblast-like cells being mostly at their intersection. It seems as though the top cell layer continues until the bottom of the glass support, covering the 3D structure. In case of the hexagonal multilayered scaffold, the cells have a more fragmented morphology, star-shaped, guided by the morphology of the unit structures. Here, the lateral walls are fully invaded (Figure 3b), the cells display a 3D arrangement, but not necessary a methodical one. The fracture into the thick layer of cells covering the scaffold shows a 3D biomimetic displacement of the cells.

**Figure 4:**
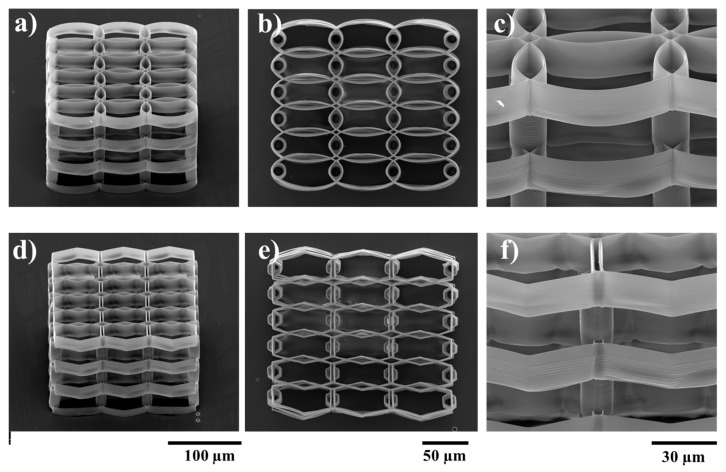
SEM (scanning electron microscopy) micrographs of ellipsoidal (upper panel) and hexagonal (lower panel) multilayered 3D structures having the layers spatially separated by cylindrical pillars. (**a**,**d**) Tilted overviews; (**b**,**e**) Top views; (**c**,**f**) Closer, tilted side views.

The above experimental studies were used to determine the optimal geometry in the XY plane for the cell growth. It is clear that the spacing between neighboring layers should be increased with respect to *Z*-axis, in order to enhance the cell migration throughout the volume of the structure. This was achieved by separating consecutive layers using appropriately placed cylindrical pillars. Each layer was designed to be 10 µm tall (~14 µm when accounting for voxel height), while pillars were designed to be 20 µm tall. The optimum overlap of consecutive rows in the XY plane was determined from previous parametrization. Pillars were placed in overlap regions for enhanced stability. Resulting structures are presented in Figure 4, for both ellipsoidal and hexagonal variants.

**Figure 5:**
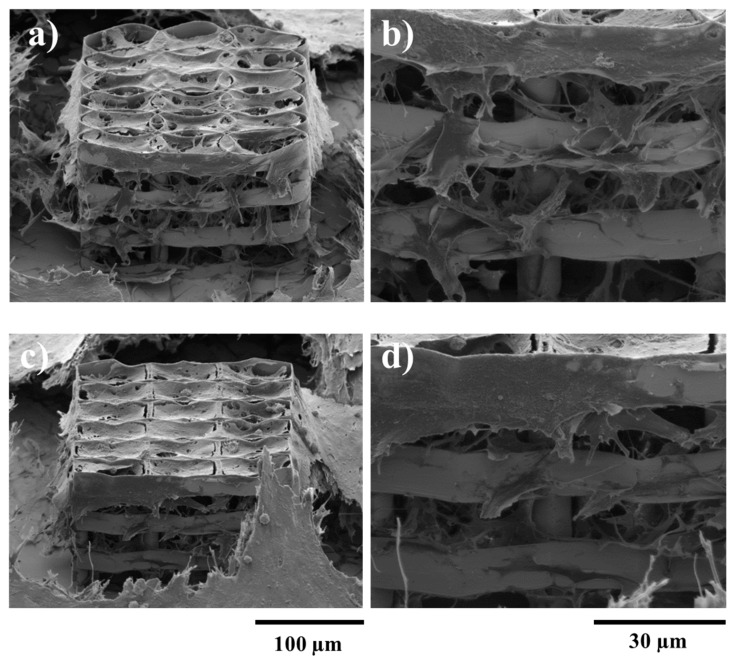
SEM (scanning electron microscopy) micrographs of MG-63 osteoblast-like cells growing on ellipsoidal (upper panel) and hexagonal (lower panel) multilayered 3D structures having the layers spatially separated by cylindrical pillars, after 7 days in cell culture. (**a**,**c**) Overviews; (**b**,**d**) Closer, tilted side view, showing cells penetration inside the structure.

To test the efficiency of this design, the structures were seeded with osteoblast-like cells and observed by SEM. It can be seen that the larger space created by the pillars between the stories of the structures allowed the cells to penetrate the interior of it. Figure 5b,d shows the 3D displacement of the cells, inside both ellipsoidal and hexagonal structures, forming a tissue-like morphology. However, the density of cells inside the structure was higher in case of the hexagonal one, as it provides more attachment points for the cells. In case of the ellipsoidal structures, the cells have a fragmented appearance, with a star-shaped morphology, for both the inside and outside of the structure. On the top wall of the structure, the cells form a fragmented layer, because the unit cylinders have a larger inner diameter, thus the cells need to stretch to have enough attachment points. In the case of the hexagonal structure, the cells on the lateral walls and inside the structure have a star-shaped morphology, but the ones on the top layer are hexagonally shaped, forming a more compact layer covering the structure. These findings are rather intriguing, considering the previous statements related to better adhesion to the elliptical shaped walls. It is highly likely that a larger top surface area in the case of elliptical shaped structure induced the collapse of the top cellular layer.

In order to find a suitable architecture for bone tissue engineering, one must take into consideration a series of factors, starting from the biocompatibility of the materials, porosity, mechanical properties and osteointegration. Considering the porosity of the structure, this not only refers to the density of pores and their dimension, which are important to allow the penetration of cells into the scaffold, as well as nutrient perfusion inside, but it can also refer to the general displacement of these pores and their ability to guide the attachment and growth of the bone cells, so that the resulting new tissue can mimic the architecture of the natural bone.

Loh et al. [29] discussed how the displacement and morphology of the pores can affect the properties and architecture of the extracellular matrix in the resulting tissue. Thus, low porosity materials initially can exhibit high proliferation rates, but compared to high porosity structures they do not allow cell differentiation [30]. However, Mandal et al. [31] proved that the proliferation of fibroblasts on porous scaffolds is facilitated not only when the dimension of the pores is higher (around 200–250 µm), but also when the dimension of pores is lower (100–150 µm) accompanied by a higher pore density. Loh et al. [29] reported data on scaffolds with various pore sizes, while the optimal dimension of pore size and density is far from being established. 3D columnar layered structures were obtained by Mata et al. [32] using microfabrication and soft lithography approaches. The structures were seeded with adult human stem cells and connective tissue progenitor cells, which showed an osteoblastic phenotype at 9 days of culturing. The cells were able to invade the interior of the structure and form colonies. Mohanti et al. [33] used a 3D printing technique to obtain layered woodpile-like structures, made of spaced polymer filaments. The porosity was varied from 20–80% and channel distances from 78–1482 µm. Also, they designed the scaffolds to exhibit both elliptical and hexagonal architecture of the pore structures. High porosity enabled a high specific surface area and thus improved the ligand density for cell attachment and spreading, as showed by the group. In our case, both elliptical and hexagonal 3D structures with optimum pore size allowed good cell attachment and proliferation with a small difference in cell density related to the top layer. Mohanti et al. [33] stated that the improved cell viability and proliferation are linked to the layered architecture of the scaffold, i.e., the network of periodic channels allowing the perfusion and mass transport inside the scaffold.

### 2.2. Structure Functionalization

**Figure 6:**
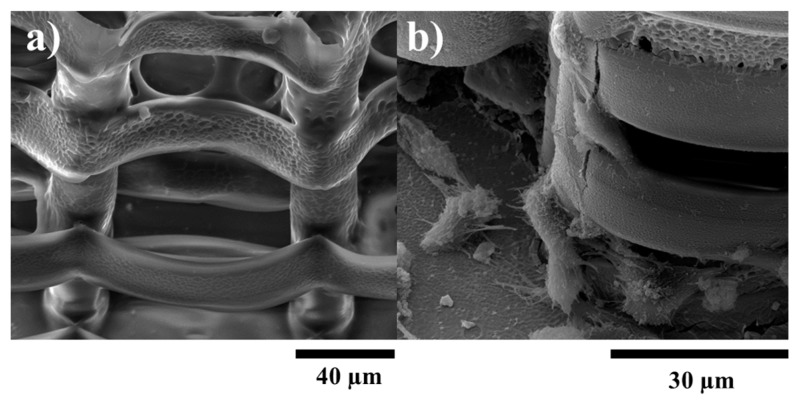
SEM (scanning electron microscopy) micrographs of: (**a**) 3D structures coated with Col-Chit-HA-MNPs (Collagen-Chitosan-Hydroxyapatite-Magnetic nanoparticles); (**b**) MG-63 cells growing on the magnetic structures.

The structures with optimized architectures were coated with Col-HA-MNPs:Chit-HA-MNPs (Collagen-Hydroxyapatite-Magnetic nanoparticles: Chitosan-Hydroxyapatite-Magnetic nanoparticles) (Figure 6). A conformal coating was relatively well achieved. Morphological investigations reveal that the coated/functionalized structure allowed the cells to attach to the inner and outer parts of the structures, in a similar fashion as for the non-coated samples.

### 2.3. Static Magnetic Field Stimulation

Previous studies on SMF simulation of the osteogenesis have mainly focused on magnetic fields above 250 M or below 50 mT. For example, Yamamoto et al. [4] reported that weak SMF between 280 and 340 mT applied to osteoblast cultures stimulated bone formation by promoting osteoblastic differentiation and/or activation. On the other hand, Cunha et al. [8] showed that increasing the magnetic field intensity up to 320 mT resulted in detrimental effects on cell proliferation and osteocalcin secretion. On the opposite, Feng et al. [6] reported that MG63 cells seeded on a PLLA discs and exposed to SMF of 400 mT showed a more differentiated phenotype. Much lower SMFs, from 50 mT down to even 3 mT, were used as biophysical stimulators of proliferation and osteoblastic differentiation of human bone marrow-derived mesenchymal stem cells [10]. Within this framework, in the present study, we cover a range of magnetic fields (100–250 mT) in between of those previously reported. In this way, we aim to explore new possibilities to optimize the cell osteogenic differentiation and to gain more insight into the roles of SMF for the stimulation of the osteogenesis.

### 2.4. Biological Assessments

Based on the above findings, the optimum design for the 3D structures was the one with ellipsoidal elements and having the layers spatially separated by cylindrical pillars (Figure 4a–c, upper panel). These structures were seeded with MG-63 osteoblast-like cells. External static magnetic fields were applied by positioning permanent magnets in the vicinity of the samples. The osteogenic effect of the magnetic structures synergizing with the static magnetic field was investigated within 30 days, by morphological evaluation, viability tests, differentiation and mineralization assays. We found evidence that the super-paramagnetic structures accelerate bone tissue formation under the external magnetic field in reference to the ones without external magnetic field.

**Figure 7:**
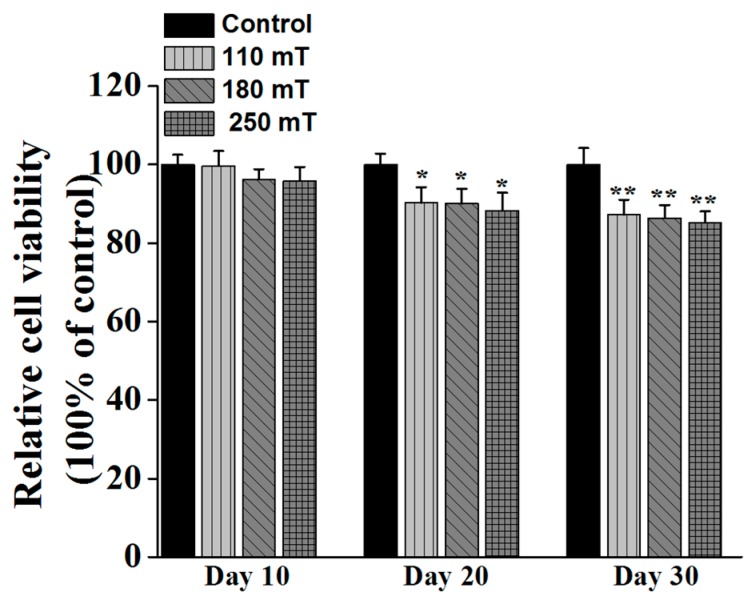
MTS viability of MG-63 osteoblast-like cells growing on ellipsoidal multilayered 3D structures having the layers spatially separated by cylindrical pillars, as a function of the applied static magnetic fields. Results for unstimulated samples (controls) are shown for comparison. Each bar represents the mean ± STD. Statistical significance was determined by Student’s *t*-test (* *p* ≤ 0.05, ** *p* ≤ 0.001).

Viability assay showed a reduced proliferation rate for the stimulated samples compared to non-stimulated ones (Figure 7). The proliferation rate was reduced with increasing magnetic field, in relation to the control i.e., unstimulated samples.

A question to be raised is why some previous studies showed stimulation of proliferation yet ours did not. Cooper [34] stated that there are three types of differentiated cells: the terminally differentiated cells that do not have any precursor left (e.g., heart cells), the cells arrested in G0, that replace death cells when needed (e.g., skin fibroblasts, smooth muscle cells, endothelial cells in blood vessels, epithelial cells in organs) and the rest of differentiated cells in organs that exhibit their function, which are not differentiating, but are replaced by stem cells undergoing differentiation (if needed). Noda [35] stated that, during the first steps of bone cell differentiation, the proliferation gene expression is supported, then the down-regulation of proliferation happens. Zhang et al. [36] used hyperoside, a flavonoid compound to study its effects on U2OS and MG63 cell lines. The group proved that the compound induces differentiation of the cells which is accompanied by cell cycle arrest in G_0_/G_1_. Whang et al. [37] showed similar results for cinnamic acid, after 7 days of culture.

In our experiments, we evaluated the proliferative activity of the MG63 cells at 4 weeks of culture, the inhibition of proliferation being associated with an advanced stage of cell differentiation. Considering the papers that we have cited, Panseri et al. [38] has evaluated the proliferation and differentiation of human osteoblast-like cells on magnetic hydroxyapatite-based scaffolds at 7, 14, and 21 days of culturing and magnetic stimulation. However, by comparing the graphs for cell proliferation measurements and ALP (Alkaline Phosphatase) measurements (differentiation), we can see that cells exhibiting higher ALP content were not undergoing proliferation anymore (this can be especially observed at day 10 and day 20). Li et al. [39] evaluated the proliferation of the cells in magnetic scaffolds just until 7 days of culturing, so these are quite early time points associated with the first steps in the differentiation process. Similar results were reported by Zheng et al. [40].

**Figure 8:**
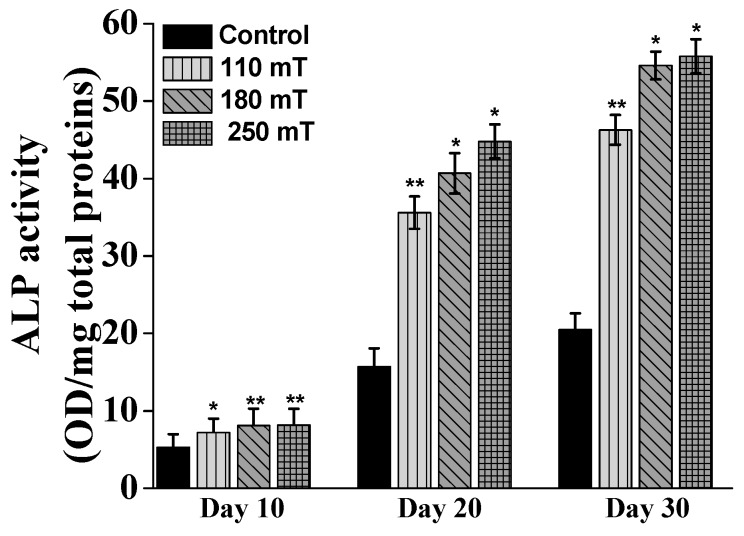
ALP (Alkaline Phosphatase) activity normalized to protein content for MG-63 osteoblast-like cells growing on ellipsoidal multilayered 3D structures having the layers spatially separated by cylindrical pillars, as a function of the applied static magnetic fields. Results for unstimulated samples (controls) are shown for comparison. Each bar represents the mean ± STD. Statistical significance was determined by Student’s *t*-test (* *p* ≤ 0.05, ** *p* ≤ 0.001).

ALP (Alkaline Phosphatase) is one of the substances in the ECM (extracellular matrix) that indicates if the osteoblast cells have entered the period of ECM development and maturation. Over the whole investigation period, the cells growing on magnetically stimulated structures produced significantly more ALP than those growing on the non-stimulated samples (Figure 8). Moreover, the ALP activity increased with increasing strength of the applied magnetic field. At 10 days of culture, the difference in ALP production for the different groups of samples did not exceed 0.3 fold, regardless the intensity of the applied static magnetic field. The difference between each group of samples became significant starting from 20 days of cells stimulation. The stimulated cells showed more than a twofold increase of ALP production compared to the control (unstimulated samples). Moreover, the ALP production increased with the intensity of the static magnetic field i.e., up to almost 3 fold for the group of samples stimulated at 250 mT. The proportion between the ALP productions for each group of samples was maintained after 30 days of magnetic stimulation, the values increasing as compared to 20 days.

**Figure 9:**
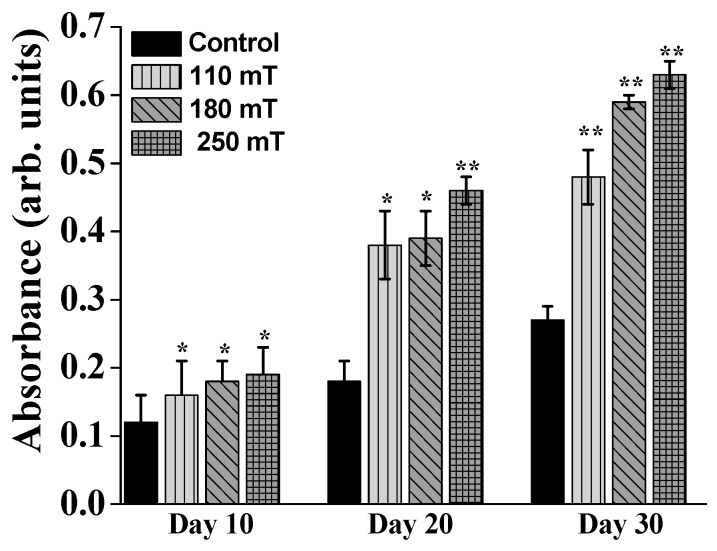
Absorbance measurements for Alizarin Red marking of the mineral deposits in MG-63 osteoblast-like cells growing on ellipsoidal multilayered 3D structures having the layers spatially separated by cylindrical pillars, as a function of the applied static magnetic fields. Results for unstimulated samples (controls) are shown for comparison. Each bar represents the mean ± STD. Statistical significance was determined by Student’s *t*-test (* *p* ≤ 0.05, ** *p* ≤ 0.001).

Alizarin Red staining was used to examine mineral deposition in the newly developed extracellular matrix. The samples exposed to magnetic fields exhibited more mineral content that the unstimulated ones (Figure 9). After 10 days of static magnetic stimulation, for the highest intensity of the magnetic field the cells exhibited an increased Alizarin Red coloring up to 0.7 fold. Similar to ALP activity measurements, the Alizarin Red dye attached more to the stimulated samples beginning with 20 days groups (almost a 2.7-fold increase in the case of the 250 mT group). The increase of Alizarin Red absorbance with stimulation time and intensity of magnetic field was more evident for the 30-day groups. These results indicate a higher number of osteoblast cells differentiating when exposed to static magnetic fields, leading to more new bone tissue formation on day 30.

**Figure 10:**
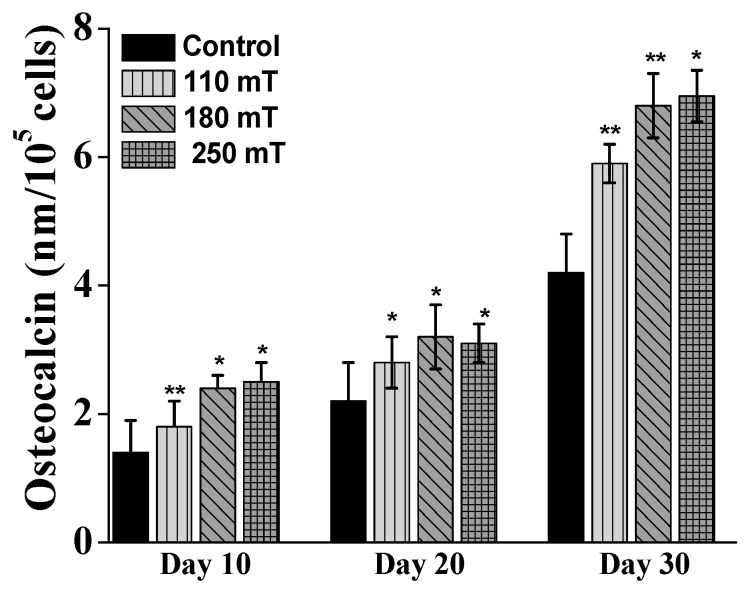
Absorbance measurements for the Osteocalcin secretion from MG-63 osteoblast-like cells growing on ellipsoidal multilayered 3D structures having the layers spatially separated by cylindrical pillars, as a function of the applied static magnetic fields. Results for unstimulated samples (controls) are shown for comparison. Each bar represents the mean ± STD. Statistical significance was determined by Student’s *t*-test (* *p* ≤ 0.05, ** *p* ≤ 0.001).

For further confirmation, the level of osteocalcin was measured using immunohistochemically staining. Osteocalcin is a bone-specific extracellular matrix protein produced by the osteoblast cells during the process of the new bone formation. At each testing time point, the samples exposed to magnetic fields exhibited higher osteocalcin formation than the unstimulated samples (Figure 10). The samples showed the highest level of osteocalcin production on the day 30 (almost a 0.7-fold increase compared to controls). The level of osteocalcin secretion increased with increasing intensity of the applied magnetic field.

All evaluated responses (cell viability, ALP, Alizarin Red staining and Osteocalcin secretion) with respect to magnetic field stimulation were statistically significant as compared with the controls (unstimulated) samples.

Our study followed the effects of static magnetic stimulation of osteoblast-like cells cultured on 3D biomimetic structures during 30 days of stimulation and incubation under standard conditions of humidity and temperature. The results showed a decreased cell proliferation in stimulated samples compared to non-stimulated samples, as measured by MTT tetrazolium salt viability assay at 30 days. This can be explained by the fact that the cells undergoing differentiation do not proliferate anymore and thus have a reduced metabolic activity [41,42]. The other results showed a more than 2-fold increase of ALP production and Alizarin Red coloring of the mineral depositions in the 250 mT sample group. These results were supported by the Osteocalcin level measurements, suggesting that the cells underwent differentiation, the degree and advancement being directly influenced by the intensity of the static magnetic field. All these results indicate that the innovative magnetic structure accelerated new bone tissue formation via the synergic action of the magnetic 3D biomimetic architecture and the applied static magnetic field, emerging as a promising approach for guiding and enhancing the process of bone growth and regeneration.

Our results are consistent with previously reported studies on static magnetic field stimulated bone tissue regeneration. Previous in vitro studies on magnetic responsive scaffolds showed the stimulating effect of the static magnetic field to the proliferation and differentiation of cells. Tampieri et al. reported on porous ceramic composite made of HA and magnetite, with enhanced in vitro cell proliferation at early stage under the external magnetic field [38]. Zhou’s group fabricated a nanofibrous scaffold composed of PLA and iron oxide nanoparticles, with good biocompatibility and guided cells orientation along the fibers under the external magnetic field [39]. A weak magnetic force with intensity of 10–50 mT was reported to accelerate osteoblast differentiation, the effect being assigned to the increased phosphorylation. Porous hydroxyapatite scaffolds containing magnetic nanoparticles enhanced the in vitro osteoblast cells growth when a magnetic field was applied [39,40]. A composite of a polyester matrix magnetically functionalized with iron oxide nanoparticles showed good ability to support and enhance the osteogenic differentiation of mesenchymal stem cells [23].

Despite these positive results, the mechanism of bone cell stimulation in magnetically responsive structures is not yet understood. It was hypothesized that the MNPs generate the microdeformation of the structure under the magnetic field, providing strain stimulation to the seeded cells. The strain stimulation would activate the cells to proliferate and differentiate and form new bone tissue. The synergy effect of magnetically responsive biomimetic structures in response to the external applied magnetic field to fasten the osteogenesis may be further amplified by combining it with chemical signaling provided by growth factors and osteogenic drugs. Moreover, the magnetism of the structures can be tailored by controlling the MNPs content in the composite.

## 3. Materials and Methods

### 3.1. Structures Design

The 3D structures were aimed to mimic the shape of a typical osteoblast cell. To this end, we designed repetitive ellipsoidal and hexagonal units, organized in a multilayered architecture. We used Python (SciPy pack, Python Software Foundation, Beaverton, OR, USA) for structure generation. After all parameters were calculated, the Python script wrote .gwl files, which are specific to the equipment used for fabrication. From a computing point of view, both geometries represent ellipses. Hexagonal structures were derived from elliptic structures, as we used only 6 points on each elliptic cell to define the hexagonal element. We employed different geometries, for different purposes, in an iterative fashion. The complete structure is composed of elements of specific geometry and position, which are repeated on each axis independently. Elements on the *X*-axis are positioned with a constant distance between the centers of neighboring elements, 30 µm. In order to determine the optimal geometry, the distanced between neighboring elements on the *Y* axis is varied according to the following equation:

(1) $$C_{Y-axis}=i\cdot inc_{x}\cdot 2^{\left(\frac{i}{10}\right)}+const$$

where $C_{Y-axis}$ represents the center of the specified element, i is the row number, $inc_{x}$ is the distance between the centers of the first two rows of elements, and const is an arbitrary constant used for positioning the whole structure with respect to the 0 position. This results in an exponential variation of the distance between the centers of neighboring elements. In other words, the overlap of elements varies exponentially with respect to the *Y*-axis. The $2^{\left(i/10\right)}$ term is used to adjust the exponential overlap to the overall size of the structure. Element diameters were 40 µm on *X*-axis and 80 µm on the *Y*-axis, respectively. The height of each layer was designed to be 20 µm (~24 µm when accounting for the voxel height). Neighboring layers intertwine for higher structural resistance. The overlap is ~8 µm depth-wise for two consecutive layers. Moreover, the layers are dislocated to the left and right, consecutively, by half the diameter of a cell on the *X*-axis. In order to compensate for this dislocation, even-numbered layers are comprised of 6 cells on the *X*-axis, while odd-numbered layers are comprised of 5 cells on the *X*-axis. After determining the optimal distance between centers of neighboring cells on each axis, we fabricated structures with fixed overlap in the *X* and *Y* directions. Height optimization is done after experimental results for optimized XY geometry.

### 3.2. Structure Fabrication and Characterization

Structure fabrication was achieved using the Photonic Professional 3D Lithography system from Nanoscribe GmbH (Eggenstein-Leopolds­hafen, Germany). This installation relies on two-photon polymerization to create 3D structures by laser direct writing (LDW by TPP). We used IP-780 as the material of choice for the structures. This material is a liquid photoresist that results in a biocompatible polymer after TPP-induced chain reaction and development. This formulation is optimized for high sensitivity in the case of rapid 3D structuring. Laser irradiation was achieved with 150 fs pulses with an 80 MHz repetition rate and centered on λ = 780 nm. The light was focused with a 63× microscope objective. The positioning was made using a hybrid system comprised of motorized and piezoelectric stages. Coarse positioning was achieved with the motorized stages, while the laser writing was done using the piezoelectric stages. Each sample was prepared by drop-casting the photoresist on a 170 µm thick glass substrate, previously cleaned by ultrasonication for 30 min in ethanol. It was then inserted into the positioning system and the laser was focused on the surface. The structure was written in the polymer drop while maintaining contact points to the substrate for adherence. The polymer was formed rapidly after irradiation without the need for additional processing. After the laser writing, the sample was taken out and immersed in Propylene Glycol Mono-methyl Ether Acetate (PGMEA) solvent for up to 15 min in order to remove the non-photopolymerized material. After removing the samples from the solvent, they were allowed to dry, in air, at room temperature.

Bare iron oxide nanoparticles (MNPs) with physical dimension of 4–20 nm have been obtained using a modified chemical co-precipitation as described in [43]. It has been proved that iron oxide nanoparticles express super-paramagnetic behavior that is preserved when incorporated in nanocomposite materials [19]. Collagen (Col), chitosan (Chit) and hydroxyapatite (HA) were acquired from Sigma Aldrich, St. Louis, MO, USA. The 3D structures were coated by spin coating at 6000 rpm with solutions containing 2 wt % chitosan, 2 wt % collagen, 2 wt % HA, and 4 wt % MNPs. Preliminary studies highlighted the differences in cell viability, density and morphology as a function of the Col:Chit ratio in the mixture. Specifically, the cell viability and density were higher for higher Col concentration, decreasing progressively with increasing Chit content. Also, in the case of compounds with a higher concentration of Col, the cells retained their native morphology, while on structures with higher Chit content, cell morphology was altered, showing specific signs of apoptosis. Based on these preliminary biological assessments, the optimal composition of the Col:Chit nanocomposite was established to be 80:20.

#### SEM

The structures were investigated by Scanning Electron Microscopy (SEM, FEI InspectS model, Hillsboro, OR, USA). Prior to SEM examination, the samples were coated with ~10 nm gold. After the cell-seeding, the samples were fixed and dehydrated using the protocol described in the next section.

### 3.3. Biological Assessments

#### 3.3.1. Cell Cultures

MG-63 osteoblast-like cells, were purchased from the European Collection of Cell Cultures (ECACC, Salisbury, United Kingdom). The cells were cultured in MEM growth medium (Biochrom, Berlin, Germany), supplemented with 10% fetal bovine serum (FBS, Biochrom), 2 mML-glutamine (Biochrom), 1% (*v*/*v*) non-essential amino-acids and 100 IU/mL of penicillin/streptomycin (Biochrom) under standard conditions of temperature and humidity (37 °C, 5% CO_2_). When confluent, the cells were detached with 1% Trypsin and seeded onto the UV-sterilized structures (5000 cells/structure) and cultured under standard conditions for 4 weeks. Before preparation for MTS assay, ALP production measurement, Alizarin Red staining and Human Osteocalcin Immunoassay, the samples were checked under an Axio Imager 2, Zeiss microscope with AxioCam MRm camera and the cells surrounding the structures were removed using a cell scraper (TPP, Trasadingen, Switzerland).

#### 3.3.2. Cells Morphological Investigations by SEM

After being cultured for 7 days under standard conditions on the 3D structures and controls, the cells were gently washed with PBS and fixed with 2.5% glutaraldehyde in PBS, during 1 h, at room temperature. After this, the cells were washed again and proceeded to the dehydration procedure. First, the samples were dehydrated in ethanol (EtOH) solutions with the indicated concentrations (2 times, during 15 min wash with Et OH 70%, 90%, respectively 100%). Then, the samples were immersed in EtOH-HMDS solutions (50%:50%; 25%:75%, respectively 0%:100% ratios, 2 times, during 3 min). Finally, the samples were let to dry prior to SEM analysis.

#### 3.3.3. MTS

5000 cells/ sample were cultured in complete MEM for 4 weeks under standard conditions of temperature and humidity. After this time, the culture medium was replaced with 16.67% MTS (Cell Titer 96^®^ Aqueous One Solution Cell Proliferation Assay, Promega, Madison, WI, USA) and 83.33% MEM (5% FBS). After 2–3 h of incubation, the supernatant was collected and 100 µL from each sample was distributed in a 96-well plate. The absorbance was measured at 490 nm, using the Mitras LB 940 (Berthold Technologies, Bad Wildbad, Germany) spectrophotometer. The viability was calculated as percent from controls (non-stimulated samples i.e., 0 mT).

#### 3.3.4. ALP

Alkaline Phosphatase production was spectrophotometrically measured at 405 nm, using the Alkaline Phosphatase Assay Kit (Colorimetric) (ab83369) (Abcam, Cambridge, UK), which uses p-Nitrophenyl Phosphate Liquid Substrate (pNPP) for cell lysate. For this, the cells were cultured similarly as for MTS. The Assay standards were prepared as following: 40 µL pNPP 5mM liquid standard solution were mixed with 160 µL Assay Buffer and serial dilution were further prepared (0, 4, 8, 12, 16, 20 nmol/well of pNPP). For the sample preparation, the cells were harvested by trypsinisation, gently washed for several times with cold PBS and resuspended in 100 µL Assay Buffer and were then centrifuged at 7000 rpm, for 15 min, to remove the insoluble components. The supernatant was transferred into 96 well-plates (100 µL/well) and completed with 50 µL of 5mM pNPP solution; 10 µL of ALP enzyme solution was only added into the standard wells. All standards and samples were incubated in the dark, at room temperature, for 60 min. Next, 20 µL of Stop Solution was added into each well, standards and samples and the absorbance was measured at 405 nm using the Mithras (Berthold Technologies) spectrophotometer. The results were expressed as units per milligram of protein in cell lysate, where the protein was assayed by the Bradford (B6916, Sigma Aldrich) method, using serum bovine albumin as standard.

#### 3.3.5. Osteocalcin

In order to measure the Human Osteocalcin protein, the cells were seeded similarly as for SE; the samples were prepared using Quantikine^®^ELISA Human Osteocalcin Immunoassay (Catalog Number DSTCN0 (R&D SYSTEMS, Minneapolis, MN, USA)), according to the producer’s specifications. The standard Osteocalcin solution in the kit was used in order to obtain a standard curve for Osteocalcin calibration. A total of 50 µL from the supernatant of each sample was added to a 96-well plate, together with 100 µL of Assay Diluent. The samples were incubated while shaking during 2 h; after this time, they were washed 3 times using the washing buffer; 200 µL from the conjugate were added in each well. After another 2 h of shaking at room temperature, the samples were washed 4 times using the washing buffer; 200 µL from the Substrate solution was added to each well and then allowed to incubate for 30 min, in the dark. At the end of this period, the reaction was finished using 50 µL of the Stop solution. The osteocalcin protein secretion was measured spectrophotometrically at 450 nm with a correction at 570 nm, using the Mitras LB 940 (Berthold Technologies).

#### 3.3.6. Alizarin Red (ARS) Assay

For mineral distribution evaluation, the cells were seeded similarly as for SEM. After 4 weeks of incubation, the samples were washed twice with double-distilled water; after this 1 mL of 40 mM ARS (pH 4.1) was added to each well. Following this, the samples were incubated for 20 min, at room temperature and then washed several times with double-distilled water while shaking for 5 min. The quantification of mineralization was done by extracting the calcified mineral at low pH, followed by neutralization with ammonium hydroxide and absorbance measurement at 405 nm.

#### 3.3.7. Statistical Analysis

The values were presented as mean ± STD (standard deviation) of 3 measurements. The data were analyzed statistically using a two-tailed Student’s test, where *p* values ≤ 0.05 were accepted as statistically significant. Each data point in the relative cell viability, ALP, Alizarin and Osteocalcin estimations was calculated as the mean of 3 different measurements performed in 3 different experiments. The standard deviation was shown as an error bar. The calculated probability that resulted in significant differences from the control samples was calculated based on the t-statistic of the variance of differences between individual observations as related to control. We defined * *p* < 0.05 and ** *p* < 0.001.

#### 3.3.8. Static Magnetic Fields Stimulation

The samples were positioned in the vicinity of gold-plated cubic Neodymium magnets (5 mm). The strengths of the magnetic fields were measured using a Phywe digital teslameter with tangential and axial Hall probes. For obtaining strengths of the magnetic field between 100 and 250 mT, we employed 1 to 3 magnets positioned in particular configurations. The Petri dishes containing the cell-seeded structures were placed on top of the magnets.

## 4. Conclusions

We demonstrated the synergistic effect of 3D magnetic structures on enhancing cell differentiation in response to static magnetic fields. We fabricated innovative, complex 3D structures of ellipsoidal and hexagonal repetitive units that mimic the native shape of osteoblast-like cells, by LDW via TPP of IP-L780 photoresist. This is the first report in the literature of the use of this technique in obtaining biomimetic 3D structures for bone tissue engineering, with this specific architectural design. The structures were coated with a magnetic composite made of collagen, chitosan, HA and MNPs. The nanoparticles provided the structures with magnetization ability suitable for cell guiding in the vicinity and inside the structure, as well as for cells differentiation and mineralization. The static magnetic field applied to the 3D structures accelerated the cell differentiation in vitro, in relation to the structures without stimulation using a magnetic field. Moreover, the cells exposed to the most intense magnetic field reached the end of proliferation period and started their differentiation faster than those in the other samples. Thus, we have succeeded in obtaining novel 3D biomimetic structures with potential for bone tissue engineering that are specifically designed to offer the best architectural features that enable the support and growth of osteoblast cells. Moreover, adding magnetic properties to the structures via the nanocomposite biocompatible coating made of collagen, chitosan, hydroxiapatite and MNPs, accelerated cell differentiation, with the potential to promote earlier development of new bone. These results provide encouraging perspectives for pre-clinical applications and set the basis for further research in developing clinically available smart engineered materials for the management of bone injures.
